# The Anticancer Efficacy of Plasma-Oxidized Saline (POS) in the Ehrlich Ascites Carcinoma Model In Vitro and In Vivo

**DOI:** 10.3390/biomedicines9080932

**Published:** 2021-07-31

**Authors:** Walison Augusto Silva Brito, Eric Freund, Thiago Daniel Henrique do Nascimento, Gabriella Pasqual-Melo, Larissa Juliani Sanches, Joyce Hellen Ribeiro Dionísio, William Capellari Fumegali, Lea Miebach, Alessandra Lourenço Cecchini, Sander Bekeschus

**Affiliations:** 1 ZIK plasmatis, Leibniz Institute for Plasma Science and Technology (INP), Felix-Hausdorff-Str. 2, 17489 Greifswald, Germany; walison.brito@inp-greifswald.de (W.A.S.B.); eric.freund@inp-greifswald.de (E.F.); gabriella.pasqual-melo@inp-greifswald.de (G.P.-M.); lea.miebach@inp-greifswald.de (L.M.); 2 Department of General Pathology, State University of Londrina, Rodovia Celso Garcia Cid, Londrina 86051-990, Brazil; thiagohenrique_d_n@hotmail.com (T.D.H.d.N.); larisanches_89@hotmail.com (L.J.S.); joyce.ribeirod@gmail.com (J.H.R.D.); william.capellari@hotmail.com (W.C.F.); alcecchini@uel.br (A.L.C.); 3 Department of General, Visceral, Thoracic and Vascular Surgery, Sauerbruchstr. DZ7, Greifswald University Medical Center, 17475 Greifswald, Germany

**Keywords:** cold physical plasma, EAC, kINPen, oncology, plasma medicine, ROS

## Abstract

Cold physical plasma, a partially ionized gas rich in reactive oxygen species (ROS), is receiving increasing interest as a novel anticancer agent via two modes. The first involves its application to cells and tissues directly, while the second uses physical plasma-derived ROS to oxidize liquids. Saline is a clinically accepted liquid, and here we explored the suitability of plasma-oxidized saline (POS) as anticancer agent technology in vitro and in vivo using the Ehrlich Ascites Carcinoma (EAC) model. EAC mainly grows as a suspension in the peritoneal cavity of mice, making this model ideally suited to test POS as a putative agent against peritoneal carcinomatosis frequently observed with colon, pancreas, and ovarium metastasis. Five POS injections led to a reduction of the tumor burden in vivo as well as in a decline of EAC cell growth and an arrest in metabolic activity ex vivo. The treatment was accompanied by a decreased antioxidant capacity of Ehrlich tumor cells and increased lipid oxidation in the ascites supernatants, while no other side effects were observed. Oxaliplatin and hydrogen peroxide were used as controls and mediated better and worse outcomes, respectively, with the former but not the latter inducing profound changes in the inflammatory milieu among 13 different cytokines investigated in ascites fluid. Modulation of inflammation in the POS group was modest but significant. These results promote POS as a promising candidate for targeting peritoneal carcinomatosis and malignant ascites and suggest EAC to be a suitable and convenient model for analyzing innovative POS approaches and combination therapies.

## 1. Introduction

Despite global efforts, cancer continues to be a devastating disease, motivating new research lines for therapy. Especially tumors of the peritoneal cavity are frequently lethal, as these metastases provoke organ dysfunction and further systemic tumor dissemination [1]. This is especially pronounced in colon, pancreatic, gastric, and ovarian cancer. Current strategies targeting peritoneal carcinomatosis (PC) involve peritoneal lavage with pre-heated liquids that contain tumor-toxic agents, a procedure called hyperthermal intraperitoneal chemotherapy (HIPEC), which often is combined with cytoreductive surgery [2]. HIPEC, however, often demonstrates severe side effects at appropriate to modest antitumor efficacy [3]. Combining HIPEC with other tumor-toxic agents might increase therapeutic efficacy while decreasing the chemotherapeutic concentration needed to elicit clinical responses. This, in turn, would also decrease side effects often observed by systemic dissemination of the chemotherapeutic agents provoked by the rapid uptake of the lavage fluid via the peritoneum.

Reactive oxygen species (ROS) have been proposed as a tumor-toxic modality for long because they often target different cell death-signaling modalities compared to anticancer drugs, provoking additive or even synergistic tumor-toxic effects [4]. However, ROS-producing modalities are limited to either single entities or treatment modalities that are complex to apply in the peritoneal cavity, such as photodynamic therapy. Recent technological leap innovations led to the emergence of a new anticancer modality, cold physical. This partially ionized gas is usually operated at body temperature and generates a plethora of ROS in the gas phase simultaneously, such as hydroxyl radicals, superoxide radicals, atomic oxygen, peroxynitrite, and singlet delta oxygen [5]. Applied in appropriate amounts, this physical modality is frequently reported to mediate tumor-toxic effects in several cancer models. Using, for instance, the kINPen plasma atmospheric pressure technology, we have provided evidence of direct anti-melanoma action in vitro [6], in vivo [7], and in clinical samples from stage IV melanoma patients ex vivo [8]. Interestingly, the complex ROS mixture of cold physical plasmas can also be applied to liquids, and these oxidized liquids can be subsequently used as a therapeutic modality. We have recently screened several types of clinically accredited liquids and identified 0.9% sodium chloride (saline) to be a suitable anticancer agent following exposure to cold physical plasma [9].

To explore the performance of kINPen argon plasma-oxidized saline (POS) as a putative anticancer agent, we used the Ehrlich Ascites Carcinoma (EAC) model and repeated intraperitoneal administration of POS for analyzing ascites cells and supernatants. We found POS to have a potent antitumor capacity while not inducing any side effects or overshooting inflammatory responses in the peritoneal cavity.

## 2. Materials and Methods

### 2.1. Generation of Cold Physical Plasma-Oxidized Saline (POS)

For generating plasma-oxidized saline (POS), 20 mL of clinical-grade isotonic sodium chloride solution (0.9% saline; Thermo Fisher Scientific, Bremen, Germany) was exposed to the gas plasma effluent of the atmospheric pressure plasma jet kINPen MED (neoplas MED, Greifswald, Germany) in a 50 mL glass beaker at a treatment distance of 20 mm as described previously [10]. The technical properties of the argon plasma jet have been outlined before [11]. As feed gas, argon gas (purity 99.999%; Air Liquide, Paris, France) was used, running at five standard liters per minute. After the treatment time of 60 min, evaporated liquid was compensated for using a predetermined amount of double-distilled water to re-establish iso-osmolarity within the POS. The solution was freshly prepared before each application.

### 2.2. Analysis of Plasma-Oxidized Saline (POS)

The pH level was measured using a calibrated pH meter (Mettler Toledo, Columbus, USA). Generation of hydrogen peroxide (H_2_O_2_) in gas plasma oxidized saline was quantified using the Amplex Ultra Red assay (Thermo Fisher Scientific, Bremen, Germany) as described before [12]. The amount of nitrite (NO_2_^−^) and nitrate (NO_3_^−^) was determined using the Griess assay (Cayman Chemical, Ann Harbor, MI, USA) according to the manufacturer’s instructions. Absorbance was measured at 540 nm 15 min after treatment using a multimode plate reader (F200; Tecan, Männedorf, Switzerland). The redox-sensitive probes 3′-(p-aminophenyl) fluorescein (APF; Thermo Fisher Scientific, Bremen, Germany) and 3′-(p-hydroxyphenyl) fluorescein (HPF; Thermo Fisher Scientific, Bremen, Germany) were used to analyze the generation of peroxynitrite (^−^ONOO), hydroxyl radical (^●^OH) and/or hypochlorous acid (HOCl). While both dyes can be oxidized by ^−^ONOO and ^●^OH, only APF is also sensitive to HOCl [13]. Both probes were added to POS at a concentration of 5 µM after treatment. Fluorescence was acquired using a multiplate reader at λ_ex_ 485 nm and λ_em_ 535 nm (M200; Tecan, Männedorf, Switzerland). To quantify any HOCl deposition in more detail, the taurine-chloramine assay was performed as described before [7]. Briefly, a taurine buffer was diluted at 1:100 in gas plasma oxidized saline. Then, a developer solution containing sodium acetate, sodium iodide, and tetramethylbenzidine in dimethylformamide was added. Absorbance was measured at 645 nm. Additionally, hydroxyl radicals (^●^OH) concentration was determined using the terephthalic acid assay [14]. The fluorescent hydroxylation product resulting from the reaction of terephthalic acid with hydroxyl radical was quantified by measuring an increase in fluorescence at λ_ex_ 318 nm and λ_em_ 426 nm. Absolute concentrations of reactive species were calculated against a standard curve.

### 2.3. Ehrlich Ascites Carcinoma (EAC) Mouse Model

For the culture and treatment of Ehrlich Ascites Carcinoma (EAC) cells, male Swiss albino mice were used (25 g body weight). They were housed under standard laboratory conditions in clean plastic cages with an ad libitum supply of food and water. The experimental procedures were carried out under the guidelines of the ethics committee at the State University of Londrina/PR in Brazil (approval number: #1633.2019.88). To minimize animal suffering, the 3R principles (reduce, refine, replace) were applied. For the induction of EAC, 5 × 10^7^ cells were injected in 50 µL phosphate-buffered saline (PBS) into the mice’s peritoneal cavities. To investigate the ex vivo effect of POS on EAC cells, ascites fluid was extracted ten days post tumor inoculation. The evaluation of the in vivo efficacy of POS was done by repeated intraperitoneal injections of 1 mL of freshly prepared POS at days 2, 4, 6, 8, and 10 before they were euthanized on the following day. Several control groups were included. In addition to a group receiving untreated saline, one group received a concentration-matched (i.e., at the concentration found in POS) control of H_2_O_2_ experimentally spiked into the saline. In another control group, oxaliplatin (OXA; 0.5 mg/kg of body weight; Sigma-Aldrich, Taufkirchen, Germany) was added to saline and injected at days 5, 7, and 9. Finally, a fifth group received a combination of both OXA (at days 5, 7, and 9) and POS (at days 2, 4, 6, 8, and 10). At sacrifice, the animals were weighted, and the peritoneal ascitic fluid was collected, centrifuged at 1300× *g* for 10 min, supernatants were separated, and cells were washed three times with PBS. All samples were stored at −20 °C for further downstream processing.

### 2.4. Metabolic Activity

After explantation from mice, EAC cells were washed three times with PBS, 1 × 10^6^ EAC cells were grown into culture flasks in Dulbecco’s minimal essential medium (DMEM; Thermo Fisher Scientific, Bremen, Germany), supplemented with 1% penicillin/streptomycin, 1% glutamine, and 10% fetal bovine serum (all Sigma-Aldrich, Taufkirchen, Germany). For ex vivo treatment, 2 × 10^5^ EAC cells were seeded into a 24-well plate, performed by incubation with a 1:1 mix of DMEM and POS (or fresh saline for control groups) for 48 h. The number of adherent cells was quantified after administration of 0.5% trypan blue (Carl Roth, Karlsruhe, Germany) utilizing a Neubauer chamber. For the quantification of the percentage of metabolically active cells, the MTT (3-(4,5-dimethylthiazol-2-yl)-2,5-diphenyltetrazolium bromide) assay (Sigma-Aldrich, Taufkirchen, Germany) was used. The assay indicates the ability of metabolically active cells to reduce the tetrazolium salt MTT to formazan that has a purple color and can be quantified using light absorbance. In control conditions, the antioxidant molecules or enzymes catalase (1000 U/mL; Sigma-Aldrich, St. Louis, MO, USA,), mannitol (25 mM; Nuclear, Diadema/SP,), and cPTIO (2-4-carboxyphenyl-4,4,5,5-tetramethylimidazoline-1-oxyl-3-oxide; 20 µM; Santa Cruz Biotecnologia, Dallas, TX, USA) were added 1:1 mix of DMEM and POS into the cells.

### 2.5. Systemic Oxidative Profile

The liquid ascitic samples obtained from mice were separated into supernatant and the cellular fraction, mainly consisting of tumor cells. Subsequently, lipid peroxidation (LOOH) analysis and total antioxidant capacity (TRAP) were evaluated in supernatants as previously described [15,16]. Lipid peroxidation results were reported in relative units of light per milligram of total protein (RUL/mg total protein), while TRAP results were expressed in μM of Trolox equivalents per milligram of total protein. In addition, catalase and superoxide dismutase (SOD) activities were evaluated in cellular fractions as previously described [17]. Briefly, cells were thawed and lysed with an Ultraturrax for 40 s on ice then centrifuged at 10,000× *g* for 10 min at 4 °C. Next, supernatants were used for experiments. Results of catalase were expressed in velocity of absorbance decrease in 1 min per milligram of total protein (V_abs_/min/mg total protein). At the same time, the amount of SOD capable of inhibiting in 50% pyrogallol autoxidation was defined as a unit of enzymatic activity per milligram of total protein (U/mg total protein). An aliquot of all samples was used to determine total protein content by the Lowry technique, modified by Miller, using bovine serum albumin as standard as previously described [18,19].

### 2.6. Quantitative Immuno-Fluorescence Imaging

Lavage-derived cells were isolated and washed with phosphate-buffered saline. Afterward, 1 × 10^6^ cells were fixed with ice-cold methanol for 15 min at −20 °C on microscopy glass slides. Blocking was performed using a blocking buffer containing PBS with 5% FCS and 0.3% Triton X-100 (Sigma-Aldrich, Taufkirchen, Germany) for 60 min. For immunofluorescence imaging, the slides were incubated with antibodies targeted at cleaved caspase 3, antibodies targeting CD45 (rabbit anti-mouse; Cell Signaling Technologies, Frankfurt/M, Germany) in antibody staining buffer containing PBS with 1% bovine serum albumin (Sigma-Aldrich, Taufkirchen, Germany), and 0.3% Triton X-100 for 16 h at 4 °C. Subsequently, the samples were incubated with fluorescently labeled (Alexa Fluor 488) secondary antibodies (goat anti-rabbit; Thermo Fisher Scientific, Bremen, Germany) and incubated for 1 h at room temperature. After several washing steps, the samples were mounted with antifade mounting medium with DAPI (4′,6-diamidino-2-phenylindole at 1µM; Vector Laboratories, Burlingame, California USA). Images were acquired utilizing the Operetta CLS high-content imaging device (PerkinElmer, Hamburg, Germany), with a 20× air objective (NA = 0.4; Zeiss, Jena, Germany) using the appropriate excitation wavelength and emission filter settings. Cell segmentation was performed utilizing algorithm-based tools of the Harmony imaging and analysis software (version 4.9; PerkinElmer, Hamburg, Germany) that separated the cells according to morphological properties. It quantified the mean fluorescence intensity of the cleaved-caspase or CD45 signal inside regions added via algorithms around the nuclear signals.

### 2.7. Multiplex Cytokine Analysis

Multiplex cytokine analysis was performed using a bead-based assay (BioLegend, Koblenz, Germany) according to the vendor’s instructions. Briefly, lavage supernatants were incubated with antibody-coated beads, and mean fluorescence intensities (MFI) of each bead population (representing a single analyte) were determined using flow cytometry (CytoFLEX S; Beckman-Coulter, Krefeld, Germany). Total cytokine concentrations in picogram per milliliter were calculated against a known standard and using 5-log fitting with dedicated LEGENpPlex analysis software (version 8; BioLegend, Amsterdam, Netherlands). To optimize compact data display in a single heatmap, data were normalized against the lavage of mice receiving untreated saline as control.

### 2.8. Statistical Analysis

Several technical and biological replicates were always carried out. Graphing and statistical analysis were performed utilizing Prism software (version 9.2; GraphPad Software, San Diego, CA, USA). To detect differences between two groups (e.g., control vs. POS), two-tailed *t*-tests were used. Multiple comparisons were carried out using *t*-test or one-way or two-way analysis of variances (ANOVA). The levels of significance are indicated as *p* < 0.05 (*), *p* < 0.01 (**), and *p* < 0.001 (***).

## 3. Results

### 3.1. Chemical Composition of POS and Its Effects on the Metabolic Activity of EAC Cells Ex Vivo

**Figure 1:**
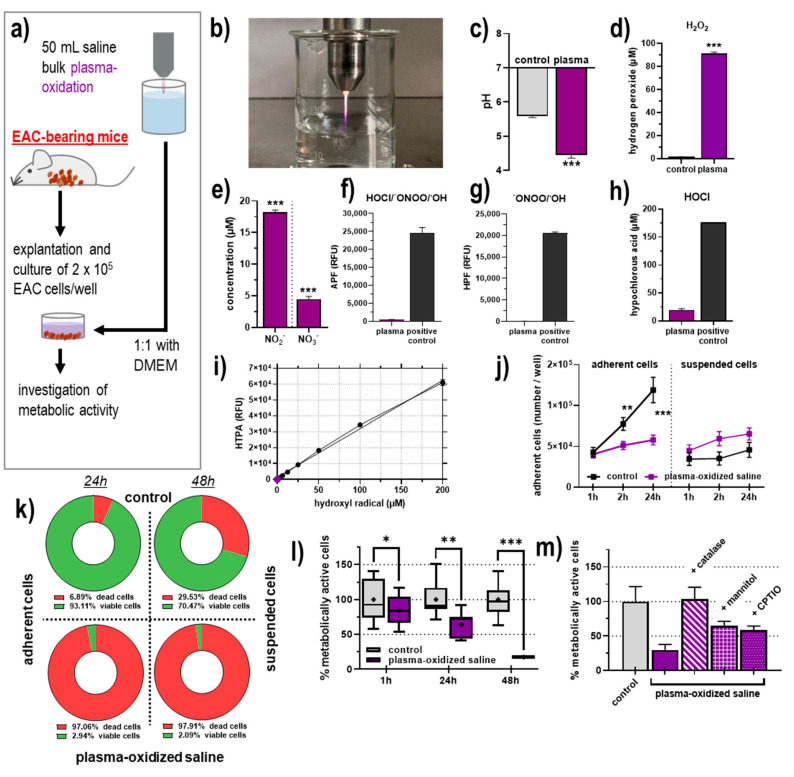
Plasma-oxidized saline reduced the metabolic activity of EAC cells ex vivo. (**a**) Schematic overview of experimental procedures; (**b**) representative image of kINPen plasma oxidation of 20 mL saline; (**c**) pH in plasma-oxidized saline; quantification of (**d**) hydrogen peroxide (H_2_O_2_), (**e**) nitrite (NO_2_^−^), and nitrate (NO_3_^−^) in plasma-oxidized saline; relative fluorescence intensity (RFU) of oxidized (**f**) APF and (**g**) HPF due to respective species in plasma-oxidized saline, direct treatment of the redox sensitive probes served as positive control for production of reactive species; quantification of (**h**) hypochlorous acid (HOCl) and (**i**) hydroxyl radical (^●^OH) in liquids, plasma treatment with HeO_2_ as feed gas was used as a positive control for HOCl production; (**j**) number of viable adherent EAC cells after explantation and incubation with either plasma-oxidized saline or untreated saline for 48 h; (**k**) percentage of adherent cells after 24 h and 48 h of POS treatment; (**l**) metabolic activity of EAC cells 1 h, 24 h, and 48 h post-incubation of plasma-oxidized or untreated saline; (**m**) metabolic activity of EAC cells 48 h after incubation with untreated or plasma-oxidized saline with the antioxidants catalase, mannitol, and cPTIO. Data are mean ± SEM of at least three independent experiments, statistical analysis was performed using *t*-test or one-way ANOVA with *p* < 0.05 (*), *p* < 0.01 (**), and *p* < 0.001 (***). EAC = Ehrlich Ascites Carcinoma; HTPA = hydroxyterephthalate; APF = fluorescence response of 3′(p-aminophenyl) fluorescein; HPF = (p-hydroxyphenyl) fluorescein.

In this work, we investigated the antitumor efficacy of plasma-oxidized saline (POS) in an Ehrlich Ascites Carcinoma (EAC) model (Figure 1a). To generate POS, the atmospheric pressure argon plasma jet kINPen MED was used (Figure 1b). The jet is known to generate large amounts of hydroxyl radicals and nitrogen radicals in the plasma gas phase, which react in liquids to a few long-lived species, for instance, hydrogen peroxide (H_2_O_2_) and nitrite (NO_2_^−^), respectively. Accordingly, the chemical composition of POS was analyzed. Upon plasma treatment, we observed a decline in pH (Figure 1c), which nevertheless was within the physiological range of medically approved saline solutions. H_2_O_2_ production is typical in POS, and deposition of H_2_O_2_ yielded a final concentration of 90 µM in POS (Figure 1d). Furthermore, reactive nitrogen species such as nitrite (NO_2_^−^) and nitrate (NO_3_^−^) were produced (Figure 1e). The two redox-sensitive probes APF (Figure 1f) and HPF (Figure 1g) were added to POS after plasma treatment to identify other species generated in PBS. Both probes are oxidized by peroxynitrite (^−^ONOO) and hydroxyl radical (^●^OH), but only APF is sensitive to hypochlorous acid. When comparing the species-dependent oxidation of both probes to direct treatment conditions, only marginal fluorescence was observed in POS, indicating a negligible production of the respective species. This was confirmed by direct quantification of hypochlorous acid (Figure 1h) and hydroxyl radical (Figure 1i) in POS.

To test the anticancer efficacy of POS, EAC cells were grown in Swiss albino mice before they were explanted from the ascites fluid, and 2 × 10^5^ cells were exposed to a mix of cell culture medium and plasma-oxidized saline (Figure 1a). The total number of adherent EAC cells was significantly greater in the groups that had received untreated saline compared to the group receiving POS when analyzed after 24 h or 48 h of incubation (Figure 1k). The number of non-adherent cells was not significantly changed in the treatment. However, an overall trend towards greater number of non-adherent cells was observed in POS conditions, suggesting apoptosis of adherent cells floating in the supernatant as non-adherent cells. Previous reports also suggested plasma treatment leading to detachment of adherent cells. To investigate whether the phenome was related to cell death or detachment, these cells were analyzed for their viability using the Trypan Blue exclusion assay. Expressed as percentages after 48 h of incubation, viability of 70% to 93% in control cells compared to only 2% in the POS-treated cells was found. These data indicated substantial toxicity of POS when applied in vitro to the cells harvested for ex vivo experiments. Moreover, the metabolic activity was analyzed and also significantly decreased after exposure to POS. This effect was modest but significant even at 1 h of incubation with POS already and increased to its most assertive extent 48 h later (Figure 1l). To confirm that reactive oxygen species (ROS) were the critical mediators of cytotoxicity, the metabolic activity was analyzed in samples having received the antioxidant enzyme catalase or the antioxidants mannitol and cPTIO. The enzyme fully abrogated cytotoxic effects, while the antioxidants partially reduced the POS-mediated toxicity in EAC cells (Figure 1m). This indicated a prominent role of the plasma-derived ROS in general and of H_2_O_2_ particularly inside the saline and suggested POS to have potent antitumor toxicity.

### 3.2. POS Reduced EAC Burden and Altered Antioxidant Capacity In Vivo

**Figure 2:**
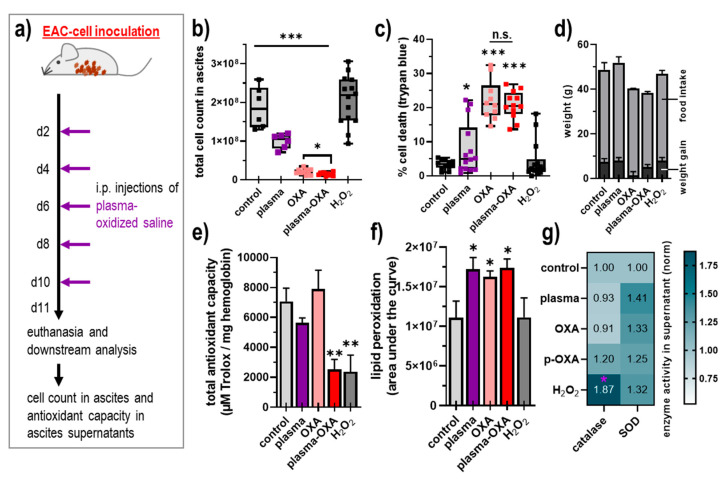
Plasma-oxidized saline reduced EAC burden and antioxidant capacity in vivo. (**a**) Schematic overview of experimental procedures; (**b**) total cell count in lavage fluids explanted from EAC-bearing mice that received injections of plasma-oxidized or untreated saline; (**c**) percentage of dead cells in the lavage; (**d**) cumulative body weight gain and total food intake of mice at day 11; (**e**) total antioxidant capacity in ascites supernatants from EAC-bearing mice that had received injections of plasma-oxidized or untreated saline; (**f**) lipid peroxidation (absolute luminescence units) in erythrocytes of the animals tested; (**g**) enzyme activity of catalase and SOD in ascites supernatants of the animals tested. Data are mean ± SEM of at least three mice per group, statistical analysis was performed using *t*-test or one-way or two-way ANOVA with *p* < 0.05 (*), *p* < 0.01 (**), and *p* < 0.001 (***) and the purple star (g) denoting *p* < 0.05 (*) compared against control. EAC = Ehrlich Ascites Carcinoma, OXA = oxaliplatin, i.p. = intraperitoneal, p(lasma)-OXA = plasma + oxaliplatin, SOD = superoxide dismutase.

To test the efficacy of POS in vivo, EAC-bearing mice received repeated injections into the peritoneal cavity (Figure 2a). The saline was left untreated, and mice were exposed to cold physical plasma, received oxaliplatin (OXA, 0.5 mg/kg body weight) alone or in combination with POS, or received a POS-concentration-matched spike-in of H_2_O_2_, resulting in a total of five treatment groups. One day after the fifth and last injection, ascites fluid was collected. The analysis of the total amounts of cells in the ascites revealed a significant reduction in the POS, OXA, and plasma-OXA but not the H_2_O_2_ group (Figure 2b). Subsequent elaboration of the percentage of viable cells showed a significant increase of dead cells in the treatment regimens of POS, OXA, and plasma-OXA applied in vivo (Figure 2c). New therapeutic approaches not only need to be effective but also safe. Accordingly, the weight and food intake of animals was monitored. While the OXA treatment showed the best antitumor capacity (Figure 2a,b), the animals also showed weight loss and less food intake, suggesting the drug to be toxic to a certain extent (Figure 2d). By contrast, the POS treatment did not induce such changes. To further explore the treatments’ effects, the cells and the supernatant were obtained from the tumor ascites. In the supernatant, the total radical-trapping antioxidant parameter (TRAP) and lipid peroxidation was analyzed. The POS and OXA treatment did not significantly alter, while it was surprising to find plasma-OXA and H_2_O_2_ to reduce the TRAP (Figure 2e) significantly. This suggests that this combination can alter the redox balance of this environment, which is often accompanied by lipid peroxidation. To analyze this possibility, the extent of lipid peroxidation was further profiled in the ascites supernatants. The plasma, OXA, and plasma-OXA groups but not H_2_O_2_ treatment were associated with significantly enhanced lipid peroxidation (Figure 2f). To understand the apparent discrepancy between reduced TRAP but unchanged lipid peroxidation levels, the enzymatic activity of two antioxidative enzymes, catalase removing H_2_O_2_, and superoxide dismutase (SOD) dismutating superoxide, was analyzed in the ascites cells. The H_2_O_2_ group showed significantly enhanced catalase activity, while all other groups did not (Figure 2g), suggesting that H_2_O_2_ spurs H_2_O_2_-focused and catalase-based antioxidant defense in the peritoneal cavity, which might affect global resources to counteract ROS, possibly resulting in decreased TRAP levels. For SOD, none of the groups showed a significant increase in its activity (Figure 2g).

### 3.3. POS Elicited Apoptosis and Modest Inflammatory Changes in the Tumor Microenvironment of the Peritoneal Cavity

**Figure 3:**
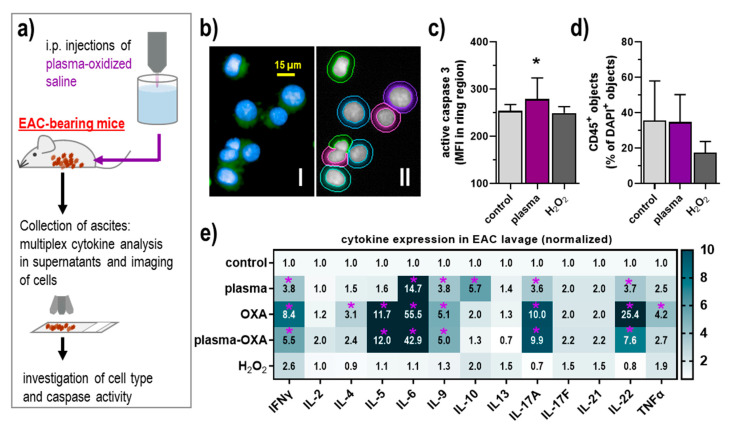
Plasma-oxidized saline showed no acute toxicity in the lavage of EAC-bearing mice while significantly altering the inflammatory profile in the peritoneal cavity. (**a**) Schematic overview of experimental procedures; (**b**) representative images of cells from lavage liquids of EAC-bearing mice with nuclear counterstaining (DAPI, blue) and active caspase 3 stainings (green), and software-based segmentation of cells; (**c**,**d**) quantification of the staining of active caspase 3 (**c**) and CD45 (**d**) in cells explanted from EAC-bearing mice that received injections of plasma-oxidized or untreated saline; (**e**) 13-plex cytokine quantification of supernatants of lavages with data normalized to controls. Data are mean + SEM of at least three mice per group, statistical analysis was performed using one-way ANOVA with *p* < 0.05 (*), or two-way ANOVA with *p* < 0.05 (*, purple) for more than three-fold changes. EAC = Ehrlich Ascites Carcinoma, OXA = oxaliplatin, i.p. = intraperitoneal, p(lasma)-OXA = plasma + oxaliplatin, IL = interleukin, IFN = interferon, TNF = tumor necrosis factor.

To investigate the tumor-toxic mode of action in vivo, the EAC cells were collected after the experiment, fixed onto microscopy slides (Figure 3a), stained for active caspase 3, and analyzed using quantitative imaging (Figure 3b). The POS but not the H_2_O_2_ treatment resulted in significantly enhanced signal intensities (Figure 3c). The analysis of tumor-infiltrating leukocytes is of great current interest when investigating the tumor microenvironment (TME). To this end, we stained the samples with anti-CD45 antibodies capable of identifying leukocytes. However, no significant differences were observed in both POS and H_2_O_2_ treatment (Figure 3d). Nevertheless, immuno-relevant changes can also occur for soluble inflammatory mediators, such as cytokines. To this end, we quantified several cytokines in the lavage supernatants of tumor-bearing mice using multiplex bead-based flow cytometry. A modest but significant increase of interleukin (IL)-6, IL-9, IL-10, IL17A, IL-22, and interferon (IFN) γ was observed for POS treatment in the EAC model (Figure 3e). Except for the lack of IL-10, OXA treatment gave a similar but much more pronounced response, with additional increases observed for IL-4, IL-5, and tumor necrosis factor (TNF) α when compared against the control. Thus, the combination of POS with OVA seemingly attenuated the levels of the significantly elevated cytokines in the OXA group for each group compared controls. For H_2_O_2_, none of the targets investigated showed a significantly changed increase or decrease compared to the control group, indicating its minor role in the local inflammatory pattern of the peritoneal TME.

## 4. Discussion

Peritoneal metastasis (PC) requires novel treatment avenues to combat cancer dissemination. Treatment with reactive oxygen species (ROS) might be a promising way of mediating anticancer effects locally and with few side effects [4]. Generating ROS-enriched liquids, e.g., via cold physical plasma treatment of medically accredited saline, is an innovative new option for such an application. Hence, we explored the anticancer efficacy and tolerability of plasma-oxidized saline (POS) in an intraperitoneal tumor model system, the Ehrlich Ascites Carcinoma (EAC).

The idea of using cold physical plasma-oxidized liquid as an anticancer tool emerged some years ago already [20]. Most studies focused on using plasma-oxidized cell culture media, the so-called plasma-activated medium (PAM). Traditionally, PAM is the directly plasma-oxidized cell culture medium, while some reports also regard in-medium-diluted POS, such as used in the experiments in Figure 1 of this study, as PAM. Several studies demonstrated that PAM could selectively eradicate cancer cells grown in 2D cultures [21] and 3D multicellular spheroids [22]. These and other studies also outlined the chemical composition of PAM, and the ROS/RNS deposited in these media might act synergistically to promote tumor cell death [23,24,25]. Mechanistically, PAM was found to affect p53 signaling [26], cause zinc liberation [27], and affect mitochondria [28]. We and others also found PAM to have additive or synergistic drugs such as complex I inhibitors [29], histone acetylase inhibitors [30], and the chemotherapeutic gemcitabine [31] that are used in the treatment of pancreatic PC of patients in the clinics [32]. In support of this, PAM reduced pancreatic tumor peritoneal metastasis in vivo [33]. Many studies using plasma-oxidized liquids implicate apoptosis as the primary mode of cell death in cancer cells [34], as also suggested in this work based on caspase 3 cleavage. Whether the extrinsic or intrinsic apoptotic pathway was activated due to in vivo application of POS in our results remains to be elucidated in future studies.

However, the limitation of PAM is that it is not accredited as a medical product because of the complex structure of cell culture medium and several hard-to-standardize components. By contrast, several other liquids such as Ringer’s lactate and NaCl are frequently used liquids in clinics [35], and both were found to be suitable for anticancer action and stability in our previous pilot study using the accredited argon plasma jet kINPen [9], making our current approach in principle eligible for case studies in patients. Previous studies found that plasma-treated Ringer’s lactate was effective against pancreatic cancer in vitro and in vivo [35], and it was reported to have a more vigorous activity than PAM [36]. Plasma-treated Ringer’s lactate was also found to be effective against other cancer entities such as ovarian cancer [37] and glioblastoma [38,39], in which a link to oxidative stress responses and altered tumor metabolism was suggested. As a redox mechanism of action, the ROS chemistry in plasma-treated Ringer’s lactate involves the generation of H_2_O_2_, which, however, depends on the concentration of lactate in a non-linear fashion [40]. Moreover, the lactate itself was suggested as an active component in these liquids [41]. Although lacking lactate, plasma-oxidized phosphate-buffered saline (PBS) was found to have high antitumor toxicity as well [42], also in 3D tumor spheroids of glioblastoma [43]. Interestingly, plasma-oxidized PBS also elicited immunogenic cancer cell death (ICD) in colorectal cancer [10] as well as in pancreatic cancer cells [44]. ICD is known to promote antitumor immunity and is a promising mode of action in both oncology and the field of plasma biomedical sciences [45]. Studies on plasma-oxidized saline in anticancer treatment, however, are scarce so far.

Similar to our findings, cPTIO—a nitric oxide scavenger—was previously found to reduce the cytotoxic effects of plasma-treated PBS on cancer cells [46]. This suggests a role of reactive nitrogen species (RNS) in addition to ROS. RNS such as nitric oxide were proposed to co-evolve from chemical reactions of POS with several enzymes at the tumor cell membrane [47]. It was interesting to note a minor but measurable amount of HOCl in the POS, which stems from cold physical plasma-derived atomic and singlet delta oxygen [48]. At pH 7, H_2_O_2_ and HOCl scavenge each other, but at pH 4.6, both species seem to co-exist to some extent, at least immediately following the physical plasma treatment of the saline solution. H_2_O_2_ is a known agent in POS chemistry, as it can trigger Fenton chemistry and ferroptosis at and in cells [49]. However, H_2_O_2_ treatment alone did not replicate the action of POS, suggesting additional effectors to be at play. Both H_2_O_2_ and POS were well-tolerated by the animals in contrast to doxorubicin. The drug is known for its side effects [50] and its anticancer efficacy. Our data on the combination of POS and oxaliplatin suggest a very modest but nevertheless significant reduction in tumor cells, which is in line with previous in vitro reports [51,52,53]. Interestingly, the combination treatment attenuated the oxaliplatin-induced inflammation in the peritoneal cavity, as observed by a decrease of, e.g., the cytokines IL-6 and IL-22 that are known to promote immuno-infiltration and activation of professional antigen-presenting cells, and to exacerbate inflammatory T-cell responses [54,55]. This suggests that POS decrease potentially toxic inflammatory side effects of oxaliplatin elicited not only in the tumor cells but also the non-malignant bystander cells in the peritoneal cavity. A reason for this might be the increased IL-10 levels seen in the POS condition, a factor observed to be released by, for instance, regulatory T cells that may have contributed to dampened inflammation [56] in the POS-OXA combination setting. The POS, OXA, and plasma-OXA conditions were also accompanied by increased levels of IFNγ, a cytokine known for its potent antitumor effects and enhancement of the antigen-presenting machinery facilitating elevated antitumor immunity [57,58]. Along similar lines, increased IL-9 levels were observed in those three conditions, pointing to a potential involvement of T_H_9 cells reported to exert antitumor activity [59]. Similarly, IL-17 is a hallmark of antitumor T_H_17 cells with profound anticancer capacity [60] and elevated IL-17 concentrations were observed in POS and OXA treatments.

In summary, it was found that POS did not cause side effects known by other chemotherapeutic agents used to treat cancer. Moreover, POS potentiated the action of OXA, decreased the antioxidant capacity of ascitic fluid, and increased lipid peroxidation that might aid in making tumor cells more susceptible to death.

## 5. Conclusions

This study suggests that our approach of combining plasma-oxidized saline with chemotherapy for targeting peritoneal carcinomatosis is promising, and further research is warranted to employ such combination therapy in a disease-specific context.

## Data Availability

The data of this study are available from the corresponding authors upon reasonable request.
